# Measurement of resection volume using computed tomography 3D modeling in lung cancer patients and survival analysis

**DOI:** 10.55730/1300-0144.6356

**Published:** 2026-05-30

**Authors:** Aykut KANKOÇ, Muhammet Tarık ASLAN, Muhammet SAYAN, Ali ÇELİK, İsmail Cüneyt KURUL, Abdullah İrfan TAŞTEPE

**Affiliations:** Department of Thoracic Surgery, Faculty of Medicine, Gazi University, Ankara, Turkiye

**Keywords:** Sublobar resection, volumetry, nonsmall cell lung cancer

## Abstract

**Background/aim:**

The prognostic significance of the volumetric extent of lung resection in nonsmall cell lung cancer remains uncertain. Three-dimensional computed tomography allows quantitative assessment of resected lung parenchyma, but the relationship between resection volume ratios and oncologic outcomes has not been clearly established. The aim in this study was to evaluate the prognostic value of three-dimensional computed tomography-derived resection volume ratios for disease-free survival after surgery for nonsmall cell lung cancer.

**Materials and methods:**

This single-center retrospective study included 141 patients who underwent anatomical resection with systematic mediastinal lymph node dissection between 2010 and 2021. Resection volume, ipsilateral lung volume, total lung volume, and derived ratios were calculated from preoperative computed tomography images. The early- and advanced-stage subgroups were analyzed separately. Receiver operating characteristic analysis was used when appropriate, whereas median values were used for exploratory survival comparisons when no meaningful threshold was identified. Disease-free survival was evaluated using Kaplan–Meier and Cox’s regression analyses.

**Results:**

In the Kaplan–Meier analysis, a higher resection-to-total lung volume ratio was associated with better disease-free survival in early-stage disease (75.6% vs. 53.4%, p = 0.015) but worse disease-free survival in advanced-stage disease (25.0% vs. 47.8%, p = 0.009). In the multivariable continuous Cox’s analysis, the resection-to-total lung volume ratio was not independently associated with disease-free survival in the early-stage patients. In contrast, in the advanced-stage patients, each 0.1 increase in this ratio was independently associated with worse disease-free survival (hazard ratio = 4.32, 95% confidence interval: 1.16–16.12, p = 0.029). No independent association was observed in the pooled multivariable model.

**Conclusion:**

The prognostic effect of the resection-to-total lung volume ratio appears to be stage-dependent in nonsmall cell lung cancer. Although favorable unadjusted associations were observed in early-stage disease, these were not confirmed in multivariable continuous analysis. In advanced-stage disease, however, higher values were independently associated with worse disease-free survival.

## 1. Introduction

Recent advances in imaging and diagnostic techniques have enabled the detection of nonsmall cell lung cancer (NSCLC) at earlier stages. As a result, the extent of anatomical resection required for optimal oncologic treatment has become a matter of renewed debate, particularly in patients with limited cardiopulmonary reserve or significant comorbidities. Parenchyma-sparing procedures have gained wider acceptance and sublobar resections are increasingly performed in selected patients. However, despite adherence to oncologic principles, their impact on long-term survival, local recurrence, and postoperative functional outcomes remains controversial.

The first randomized trial addressing this issue, LCSG 0821, demonstrated superior survival and lower local recurrence after lobectomy than after sublobar resection in stage IA NSCLC [1]. Following that study, segmentectomy was generally reserved for older patients or those with limited pulmonary function. Later institutional series and analyses based on the SEER database produced conflicting results [2,3]. More recently, two large prospective randomized trials, JCOG 0802/WJOG4607L and CALGB 140503, showed comparable overall survival (OS) and disease-free survival (DFS) after sublobar resection and lobectomy in carefully selected early-stage patients [4,5]. These findings have further intensified the debate on how much lung parenchyma should be resected to achieve oncologic adequacy without unnecessary functional loss.

At the same time, three-dimensional (3D) imaging has made it possible to quantify thoracic structures more precisely using computed tomography (CT) data. Three-dimensional volumetry has been used for tumor volume estimation [6,7], prediction of postoperative pulmonary function [8,9], donor–recipient lung matching [10], and planning of volume-reduction procedures [11]. In contrast to the conventional qualitative classification of resection type, volumetric analysis offers a quantitative measure of the actual extent of parenchymal removal. However, whether such quantitative measures are associated with postoperative oncologic outcomes in NSCLC remains unclear.

## 2. Materials and methods

This single-center retrospective cohort included consecutive patients with histologically confirmed NSCLC who underwent anatomical lung resection with systematic mediastinal lymph node dissection between January 2010 and December 2021. All cases were reclassified according to the 8th edition TNM staging system. Only patients without nodal (N0) or distant (M0) metastasis were included in the analysis. Demographic and clinical data, including age, sex, comorbidities, resection type, tumor size, histopathologic subtype, and follow-up status, were recorded. Preoperative volumetric measurements were obtained from thoracic CT scans.

Patients with conditions likely to interfere with 3D volumetric assessment were excluded. These included thoracic wall invasion; obstructive atelectasis, pneumonia, or lymphangitic spread; pleural conditions affecting lung volume such as pneumothorax, hemothorax, or pleural effusion; severe emphysematous destruction; and additional mediastinal or thoracic wall lesions occupying substantial intrathoracic space. Patients with a history of prior thoracic surgery affecting lung volume, preoperative CT performed more than 1 month before surgery, or insufficient image quality for 3D reconstruction were also excluded. Patients with unavailable follow-up data were not included.

**Figure 1:**
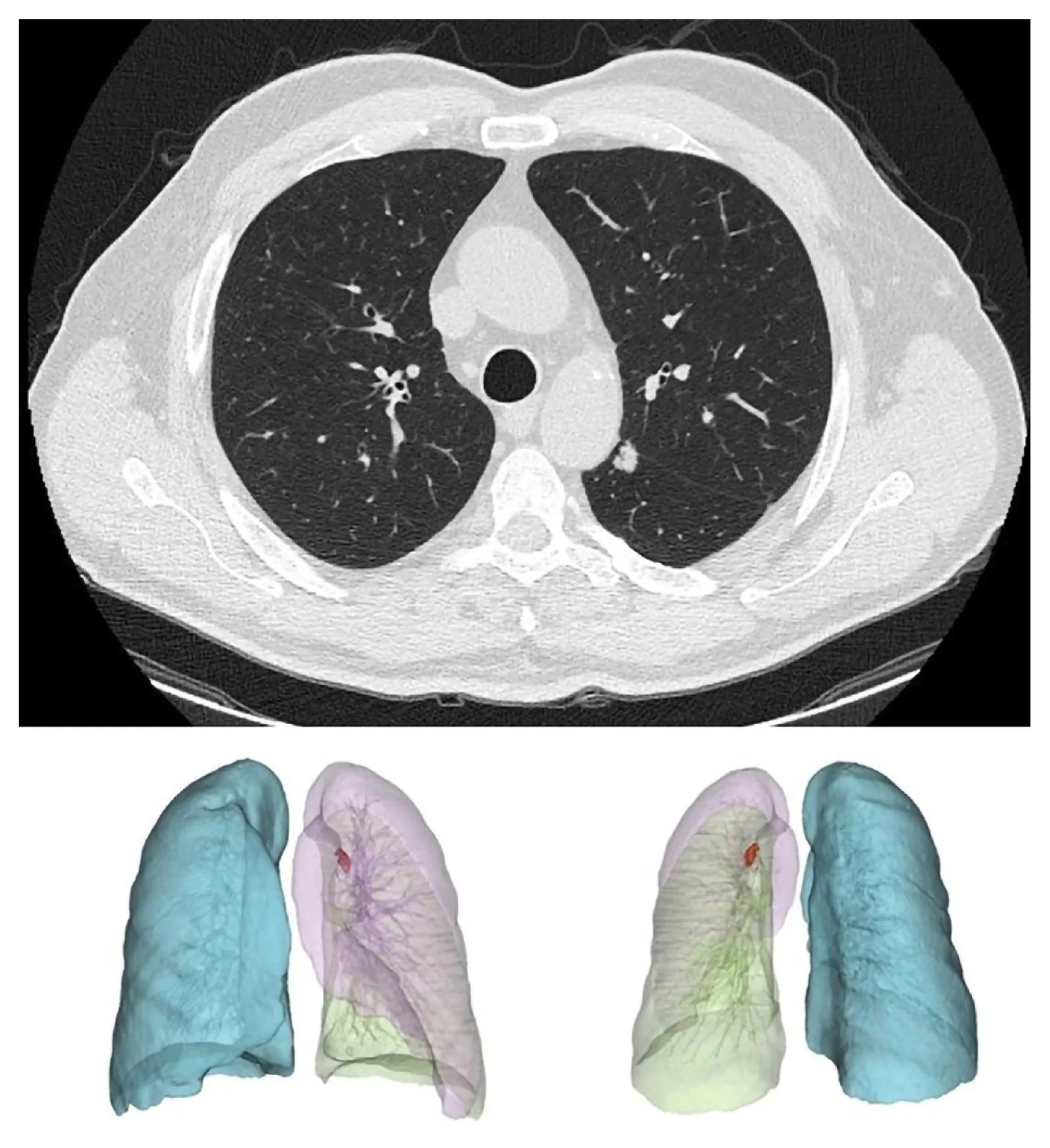
Axial section and 3D reconstruction of the CT of a patient included in the study.

Thoracic CT scans obtained within the month before surgery were used for 3D reconstruction and volumetric analysis. Three-dimensional modeling was performed using 3D Slicer software (Fedorov A, et al., Magn Reson Imaging 2012; 30: 1323-1341). Airways and vascular structures were excluded by automatic thresholding followed by manual correction by two investigators. Resection volume, ipsilateral lung volume, contralateral lung volume, total lung volume, and tumor volume were measured in cubic centimeters (Figure 1). The resection volume-to-ipsilateral lung volume ratio and the resection volume-to-total lung volume ratio were calculated for each patient.

The patients were categorized as early stage (stage I–IIA) and advanced stage (stage IIB–IIIA) according to the 8th edition TNM classification. The primary outcome of the study was DFS, defined as the interval between surgery and the first occurrence of recurrence or death. OS was defined as the interval between surgery and death from any cause. Resection extent was assessed using absolute resection volume and two proportional indices: the resection volume-to-ipsilateral lung volume ratio and the resection volume-to-total lung volume ratio. Because receiver operating characteristic analysis did not identify meaningful thresholds in all settings, median values were used as cut-off points in exploratory survival comparisons when appropriate.

Statistical analyses were performed using IBM SPSS Statistics version 23.0 (IBM Corp., Armonk, NY, USA). Continuous variables are presented as mean ± standard deviation or median (range), and categorical variables as number (percentage). The distribution of continuous variables was assessed using histograms and the Kolmogorov–Smirnov test. Between-group comparisons were performed using the independent t-test or Mann–Whitney U test, as appropriate. Categorical variables were compared using the chi-squared test or Fisher’s exact test. Correlations between volumetric variables were evaluated using Spearman’s correlation analysis. Receiver operating characteristic analysis was performed to assess the discriminatory performance of volumetric parameters for DFS. When no meaningful threshold was identified, exploratory dichotomized survival analyses were performed using median values. Survival was estimated using the Kaplan–Meier method and compared with the log-rank test. Cox’s proportional hazards regression analysis was performed to evaluate the association between volumetric parameters and DFS. In addition to dichotomized exploratory analyses, the resection volume-to-total lung volume ratio was analyzed as a continuous variable in Cox’s regression models and served as the primary basis for inferential interpretation. Because of the high collinearity between the resection volume-to-ipsilateral lung volume ratio and the resection volume-to-total lung volume ratio, these variables were not entered into the same multivariable model. All tests were two-sided and a p-value of less than 0.05 was considered statistically significant.

The study was approved by the institutional review board (approval no. E-77082166-604.01.02-534817) and was conducted in accordance with the principles of the Declaration of Helsinki.

## 3. Results

A total of 141 patients were included in the study, of whom 109 (77.3%) were male. The mean age was 63.7 ± 8.3 years. Adenocarcinoma was the most common histopathologic subtype, observed in 69 patients (48.9%). The median radiologic tumor diameter was 2.4 cm (range: 0.7–9.5 cm). Pathologic stage IA was the most frequent stage (n = 80, 56.7%) and lobectomy was the predominant resection type (n = 104, 73.8%). The baseline clinicopathologic characteristics are summarized in Table 1. The median tumor volume measured by 3D volumetric analysis was 6.31 cm^3^ (range: 0.01–523.99 cm^3^). Median ipsilateral lung volume and total lung volume were 2578.9 cm^3^ (range: 975.3–5136 cm^3^) and 5117.6 cm^3^ (range: 2272.5–9461.4 cm^3^), respectively. The median resection volume-to-ipsilateral lung volume ratio and resection volume-to-total lung volume ratio were 0.42 and 0.21, respectively.

When the patients were categorized as early stage (I–IIA) and advanced stage (IIB–IIIA), both OS and DFS differed significantly between the groups. The 5-year OS rates were 76.4% and 65.5% in the early- and advanced-stage cohorts, respectively (p = 0.016), whereas the corresponding 5-year DFS rates were 65.0% and 37.3% (p < 0.0001). In contrast, resection volume, the resection volume-to-ipsilateral lung volume ratio, and the resection volume-to-total lung volume ratio did not differ significantly between stages (p = 0.307, p = 0.182, and p = 0.342, respectively). Among the clinicopathologic variables, chronic obstructive pulmonary disease was more frequent in the early-stage group (p = 0.029), whereas histopathologic distribution showed a borderline stage-related difference (p = 0.055). No significant between-group differences were observed for sex, age category, smoking history, cardiac comorbidity, or resection type.

Spearman’s correlation analysis showed no significant association between tumor volume and the volumetric resection parameters in the overall cohort or in stage-specific analyses (all p > 0.05). In contrast, the resection volume-to-ipsilateral lung volume ratio and the resection volume-to-total lung volume ratio were strongly correlated with each other in both early- and advanced-stage subgroups (all p < 0.001), indicating marked collinearity between these two proportional indices.

Receiver operating characteristic analysis showed limited discriminatory performance of volumetric parameters for DFS in the early-stage patients and no meaningful cut-off value could be identified in this subgroup. Therefore, median values were used for exploratory survival comparisons in early-stage disease. In the advanced-stage patients, the resection volume-to-total lung volume ratio showed the strongest discriminatory performance for DFS (AUC = 0.731, p = 0.027), whereas the resection volume-to-ipsilateral lung volume ratio showed weaker, borderline discrimination. Accordingly, the resection volume-to-total lung volume ratio was selected as the primary volumetric variable for subsequent survival modeling.

**Figure 2:**
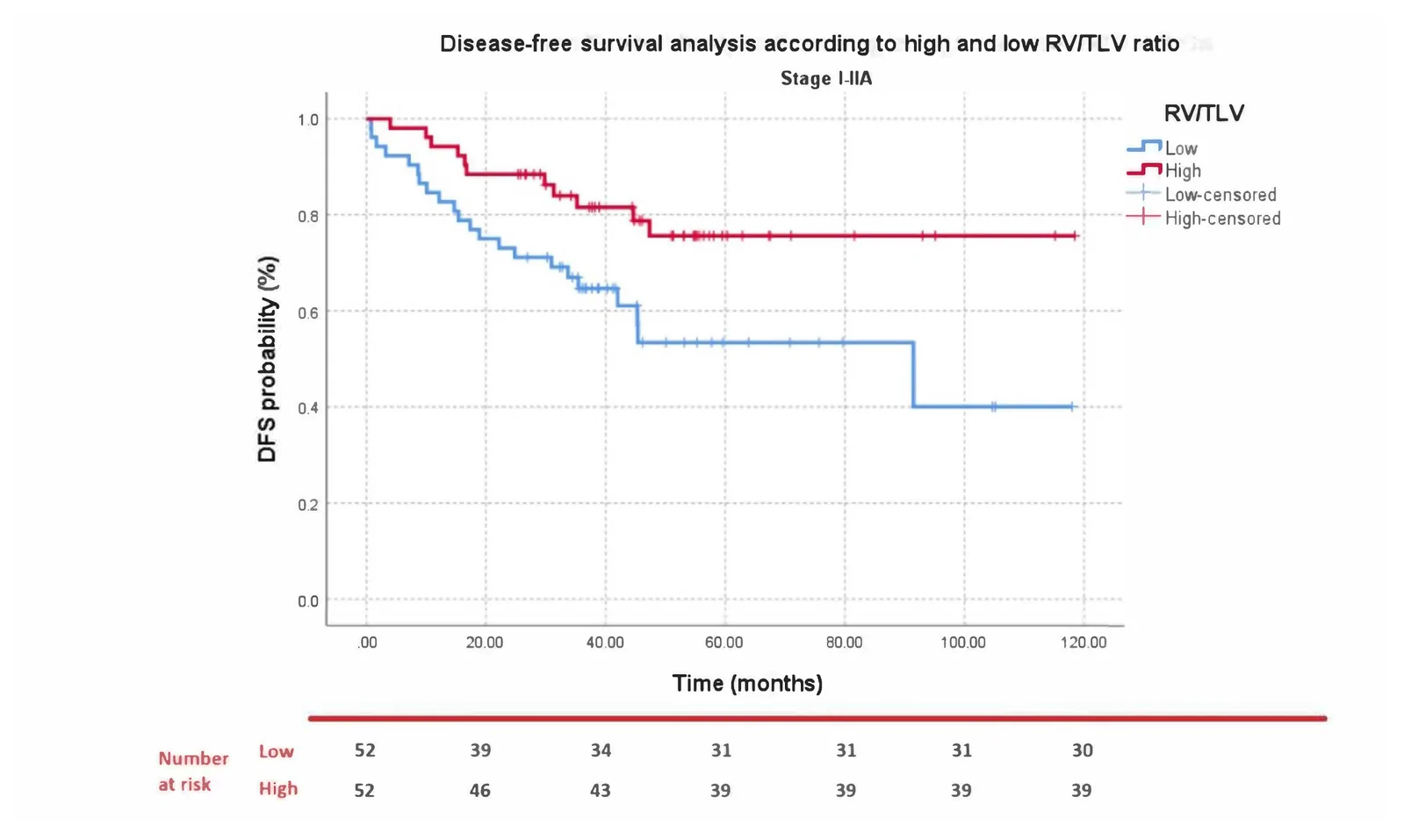
Analysis of disease-free survival in early-stage patients according to RV/TLV ratio.

**Figure 3:**
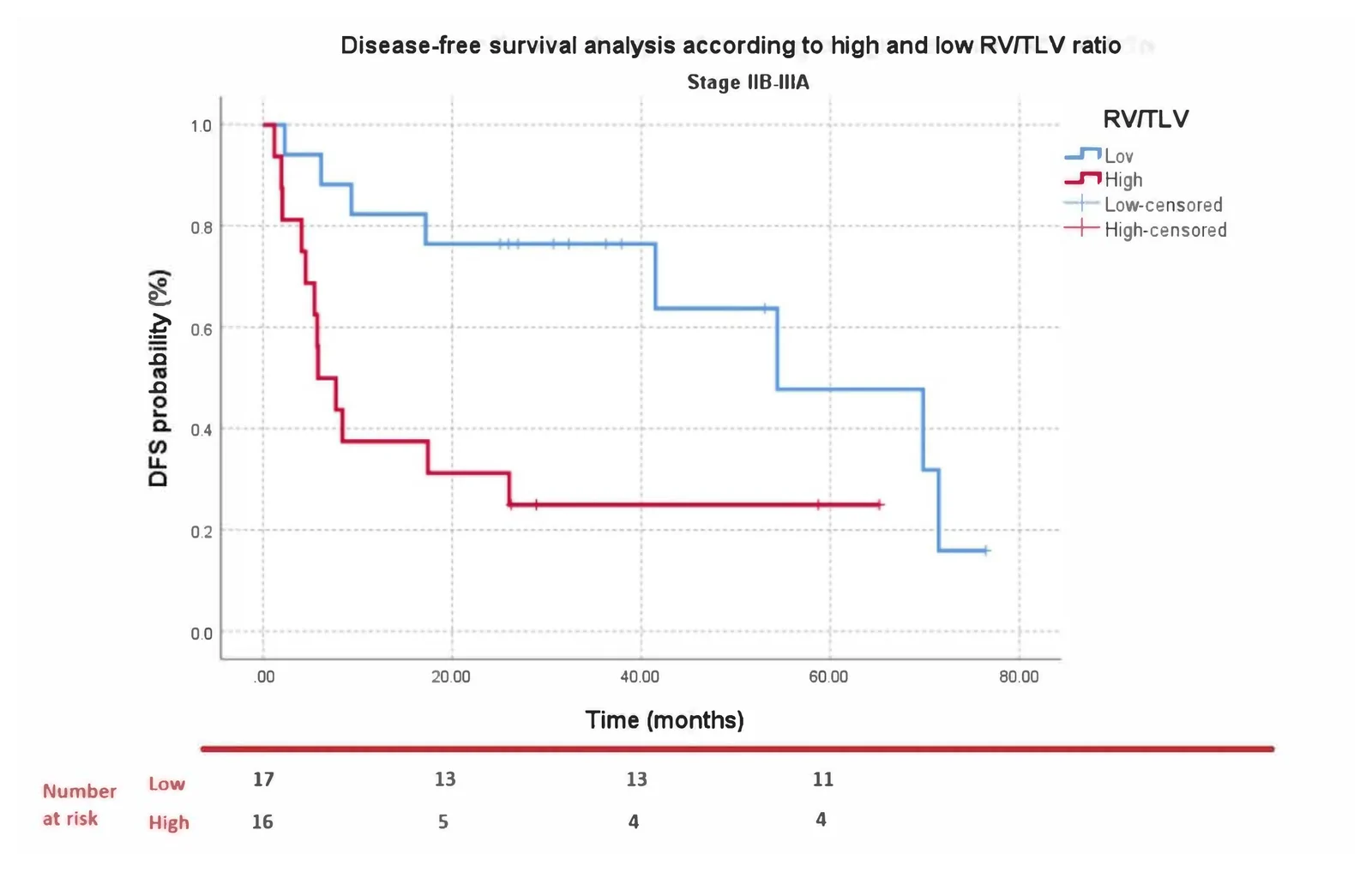
Analysis of disease-free survival in advanced-stage patients according to RV/TLV ratio.

**Figure 4:**
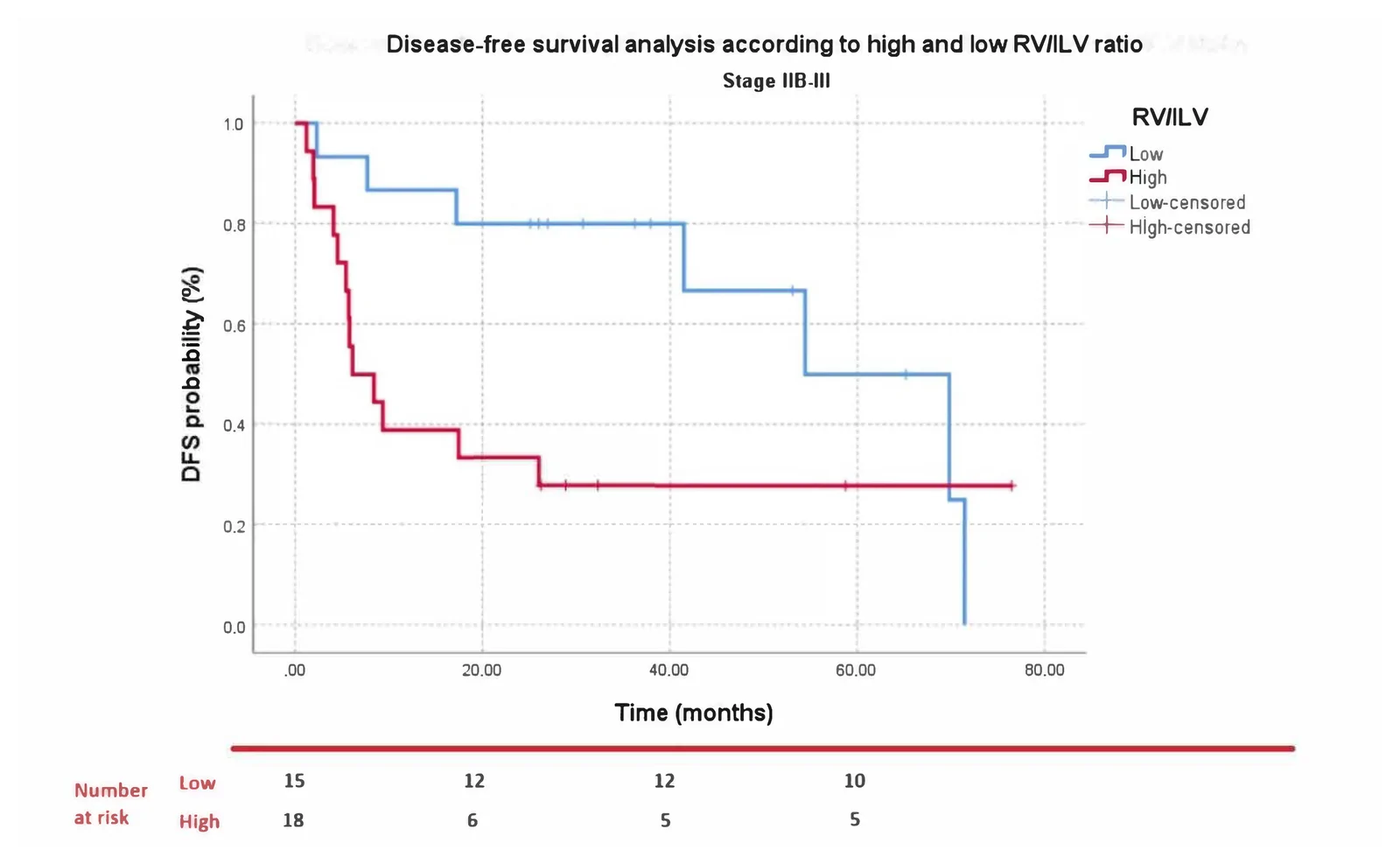
Analysis of disease-free survival in advanced-stage patients according to RV/ILV ratio.

The Kaplan–Meier analysis demonstrated a stage-dependent association between the resection volume-to-total lung volume ratio and DFS. In the early-stage patients, a higher ratio was associated with significantly better DFS (5-year DFS: 75.6% vs. 53.4%, p = 0.015) (Figure 2; Table 2). In contrast, in advanced-stage patients, a higher ratio was associated with significantly worse DFS (5-year DFS: 25.0% vs. 47.8%, p = 0.009) (Figure 3). By comparison, the resection volume-to-ipsilateral lung volume ratio showed no significant association with DFS in early-stage disease, whereas a lower ratio was associated with better DFS in advanced-stage patients (5-year DFS: 50.0% vs. 27.8%, p = 0.044) (Figure 4). Resection volume alone was not associated with DFS in either subgroup.

When analyzed as a continuous variable, the resection volume-to-total lung volume ratio was not associated with DFS in the early-stage patients in the univariable analysis (per 0.1 increase: HR = 0.83, 95% CI: 0.58–1.19, p = 0.314), and no independent association was observed after adjustment for age, resection type, or cardiac comorbidity (HR = 1.01, 95% CI: 0.61–1.69, p = 0.968) (Table 3). In contrast, in the advanced-stage patients, each 0.1 increase in the resection volume-to-total lung volume ratio was associated with worse DFS in the univariable analysis (HR = 3.40, 95% CI: 1.69–6.82, p = 0.001) and remained independently associated with worse DFS after multivariable adjustment (HR = 4.32, 95% CI: 1.16–16.12, p = 0.029).

In the pooled multivariable Cox’s model including stage, age, cardiac comorbidity, resection type, histopathology, forced expiratory volume in 1 s, forced vital capacity, and diffusing capacity of the lung for carbon monoxide, the resection volume-to-total lung volume ratio was not independently associated with DFS when analyzed as a continuous variable (per 0.1 increase: HR = 1.17, 95% CI: 0.61–2.25, p = 0.642). In this model, resection type and diffusing capacity of the lung for carbon monoxide remained independently associated with outcome.

In the subgroup restricted to stage I patients who underwent sublobar resection or lobectomy, the resection volume-to-total lung volume ratio was not significantly associated with OS or DFS (p = 0.143 and p = 0.058, respectively). OS findings were otherwise broadly consistent with the DFS analyses. The early-stage patients had significantly better OS than the advanced-stage patients (5-year OS: 76.4% vs. 65.5%, p = 0.016). In early-stage disease, a higher resection volume-to-total lung volume ratio was associated with better OS (86.2% vs. 64.9%, p = 0.011), whereas no significant association was observed in the advanced-stage patients.

## 4. Discussion

In the present study, 3D CT-derived resection volume ratios showed a stage-dependent association with DFS after anatomical resection for node-negative NSCLC. Among the volumetric parameters evaluated, the resection volume-to-total lung volume ratio provided the most consistent signal. In dichotomized survival analyses, a higher ratio was associated with more favorable DFS in early-stage disease, whereas the same finding was associated with worse DFS in advanced-stage disease. However, continuous-variable Cox’s analysis showed that this apparent favorable association in early-stage disease was not independently maintained, while the adverse prognostic effect in advanced-stage disease remained significant. These findings suggest that the prognostic meaning of volumetric resection extent is not uniform across disease stages. Accordingly, the prognostic interpretation of resection volume/total lung volume in the present study should be based primarily on the continuous-variable Cox’s models, while the dichotomized Kaplan–Meier findings should be regarded as exploratory.

The extent of parenchymal resection in early-stage NSCLC remains controversial. Sublobar resections have increasingly been performed even in patients with adequate pulmonary reserve, yet their long-term oncologic impact is still debated. Some authors have emphasized a higher risk of local recurrence after sublobar resection, particularly in stage I disease, whereas others have reported comparable overall and DFS after sublobar and lobar procedures [12,13]. The potential functional benefit of parenchyma-sparing surgery is also unclear. Another argument in favor of segmentectomy is the possibility of a second primary lung cancer after the initial resection, with an estimated incidence of approximately 3% per year, making preservation of lung parenchyma relevant for potential future surgery [14,15].

Three major prospective randomized trials have shaped this debate. LCSG 0821 demonstrated higher recurrence and inferior local control after sublobar resection than after lobectomy in tumors ≤3 cm without nodal involvement [1]. In contrast, the more recent JCOG 0802/WJOG4607L and CALGB 140503 trials showed comparable OS and DFS between sublobar resection and lobectomy in selected patients with small peripheral tumors [4,5]. However, in these studies resection extent was classified according to procedure type rather than the actual amount of lung parenchyma removed. In this context, our findings suggest that a volumetric assessment may provide additional prognostic information beyond the conventional distinction between lobectomy and sublobar resection.

In early-stage disease, dichotomized analyses indicated that a higher resection volume-to-total lung volume ratio was associated with more favorable DFS and OS. One possible explanation is that, in patients with limited tumor burden, proportionally wider anatomical resection may improve local control without being offset by the biological aggressiveness seen in more advanced disease. In this setting, volumetric extent may reflect oncologic adequacy rather than excessive parenchymal loss. However, these findings should be interpreted with caution. In the subgroup analysis restricted to stage I patients who underwent lobectomy or sublobar resection, no statistically significant difference was observed. More importantly, when the resection volume-to-total lung volume ratio was analyzed as a continuous variable in multivariable Cox’s analysis, no independent association with DFS was identified in the early-stage patients. Therefore, the favorable signal observed in unadjusted and dichotomized analyses should be regarded as exploratory and not as evidence of a confirmed independent prognostic effect.

In contrast, the prognostic pattern was reversed in advanced-stage disease. In this subgroup, a higher resection volume-to-total lung volume ratio was associated with worse DFS in the Kaplan–Meier analysis and remained independently associated with worse outcome in continuous multivariable Cox’s analysis. This finding suggests that, in more advanced tumors, a larger proportional resection may no longer represent oncologic adequacy alone, but may instead reflect greater local tumor burden and more extensive parenchymal involvement. Under these conditions, the adverse biological behavior of the disease may outweigh any potential benefit of wider resection. The persistence of this association in continuous-variable analysis strengthens the interpretation that the prognostic value of resection volume/total lung volume in advanced-stage disease is not merely a consequence of arbitrary cut-off selection.

An additional finding of the present study was that the resection volume-to-total lung volume ratio appeared to be more informative than the resection volume-to-ipsilateral lung volume ratio. This may have been because the total lung volume provides a more stable denominator and may better reflect the relative burden of resection at the whole-lung level. In contrast, the ipsilateral ratio may be more sensitive to local anatomical variation. Notably, tumor volume was not significantly correlated with the volumetric resection ratios, suggesting that these indices do not simply mirror tumor size, but rather the extent of parenchymal sacrifice within the context of surgical decision-making.

The pooled multivariable analysis further supports this interpretation. When all the patients were analyzed together and adjustment was made for stage, age, cardiac comorbidity, resection type, histopathology, pulmonary function parameters, and diffusing capacity of the lungs for carbon monoxide, resection volume/total lung volume was not independently associated with DFS. This finding is consistent with the opposite directions of effect observed in stage-specific analyses and indicates that a single linear effect across the entire cohort is unlikely. In other words, the prognostic value of this volumetric parameter appears to depend on disease stage rather than acting as a uniform predictor in all patients.

Our study has several limitations. First, its retrospective single-center design may have introduced selection bias. Second, the sample size and number of events, particularly in the stage-specific subgroup analyses, may have increased the risk of overfitting in the multivariable models and reduced the precision of some estimates. Third, although volumetric analysis provided an objective measure of resection extent, postoperative pulmonary function was not assessed; therefore, the functional consequences of greater parenchymal loss could not be evaluated. Fourth, because accurate 3D modeling required exclusion of several clinical scenarios that could distort lung volume measurements, the cohort was highly selected and may not fully reflect routine clinical populations. Finally, the study included only node-negative patients and focused on anatomical resections, which may limit the generalizability of the findings to other surgical populations.

Three-dimensional CT-derived resection volume ratios may provide prognostic information in surgically treated node-negative NSCLC. Among these parameters, the resection volume-to-total lung volume ratio showed the most consistent association with DFS. However, its prognostic effect appeared to be stage-dependent. In early-stage disease, favorable associations observed in dichotomized analyses were not confirmed in multivariable continuous analysis. In advanced-stage disease, by contrast, higher resection volume/total lung volume values remained independently associated with worse DFS. These findings suggest that quantitative assessment of resection extent may contribute to postoperative risk stratification, particularly in advanced-stage disease.

## Figures and Tables

**Table 1:** Demographic, clinical, and histopathologic characteristics of the patients.

		n	%	Mean + SD^a^	Median (min-max)
**Sex**					
	Male	109	77.3		
	Female	32	22.7		
**Age**				63.7 ± 8.25	64 (33–83)
	≥65	70	49.6	70.21 ± 3.96	70 (65–83)
	<65	71	50.4	57.28 ± 6.04	59 (33–64)
**Histopathology**					
	Adenocarcinoma	69	48.9		
	Squamous cell carcinoma	61	43.3		
	Other	11	7.9		
**Smoking history**				Package/year	Package/year
	Current	73	58.9	54.06 ± 29.64	45 (10–150)
	Former	31	25	39.3 ± 29.68	30 (1–120)
	Never	20	14.2	-	-
**Comorbidities**		101	71.6		
	Cardiac	60	42.6		
	Respiratory	21	14.9		

a **SD:** Standard deviation

**Table 2:** Five-year overall and disease-free survival rates according to volumetric parameters.

	5-year OS^d^ (%)	ES^f^ 5-year OS (%)	AS^g^ 5-year OS (%)	5-year DFS^e^ (%)	ES 5-year DFS (%)	AS 5-year DFS (%)
**General**	74	76.4	65.5	59	65	37.3
**p-values** **	**0.016**			**<0.0001**		
**RV** a						
**Low**	74.1	74	80	55.2	61.4	35.6
**High**	73.4	79	55.6	62.1	69.5	40
**p-values**	0.41	0.966	0.108	0.93	0.687	0.729
**RV/ILV** b						
**Low**	70.9	68.9	77.8	50.9	57.8	50
**High**	76.4	83.7	55.6	60.7	72.5	27.8
**p-values**	0.541	0.082	0.192	0.822	0.105	**0.044**
**RV/TLV** c						
**Low**	67	64.9	73.5	52.1	53.4	47.8
**High**	79.3	86.2	56.3	63.8	75.6	25
**p-values**	0.135	**0.011**	0.068	0.313	**0.015**	**0.009**

a RV: Resection volume,

b ILV: Ipsilateral lung volume,

c TLV: Total lung volume,

d OS: Overall survival,

e DFS: Disease-free survival,

f ES: Early stage,

g AS: Advanced stage.

** p < 0.05 (shown in bold) was considered statistically significant.

**Table 3:** Cox’s regression analysis for disease-free survival according to RV/TLV.

Stage-specific continuous Cox		HR	95% CI	p
	Early stage - univariable	0.83	0.58–1.19	0.314
	Early stage - multivariable	1.01	0.61–1.69	0.968
	Advanced stage - univariable	3.40	1.69–6.82	**0.001**
	Advanced stage - multivariable	4.32	1.16–16.1	**0.029**
**Pooled multivariable Cox**				
	RV/TLV	1.16	0.60–2.25	0.642
	Age	1.38	0.51–3.73	0.518
	Cardiac comorbidity	0.43	0.14–1.26	0.126
	Resection type	7.31	2.44–21.8	**0.001**
	Histopathology	0.92	0.42–1.99	0.839
	FEV1%	1.04	0.98–1.10	0.154
	FVC%	0.97	0.92–1.03	0.421
	DLCO%	0.95	0.92–0.98	**0.006**
	Stage	1.54	0.51–4.65	0.441

HR: Hazard ratio, CI: Confidence interval, RV/TLV: Resection volume/total lung volume, FEV1: Forced expiratory volume in 1 s, FVC: Forced vital capacity, DLCO: Diffusing capacity of the lungs for carbon monoxide. p < 0.05 (shown in bold) was considered statistically significant.
